# Algorithms in acetabular fracture classifications

**DOI:** 10.1007/s00402-024-05599-6

**Published:** 2024-10-23

**Authors:** Axel Gänsslen, Jerome Tonetti, Tim Pohlemann

**Affiliations:** 1 Department of Trauma Surgery, Hannover Medical School, Hannover, Germany; 2 University Hospital, Johannes Wesling Hospital, Minden, Germany; 3 Department of Orthopaedic and Trauma Surgery, Medical University Innsbruck, Innsbruck, Austria; 4 Department of Trauma, Hand and Reconstructive Surgery, Saarland University, Homburg/Saar, Germany

**Keywords:** Acetabular fracture, Classification, Reliability, Radiographic lines, Step-wise algorithm

## Abstract

Acetabular fractures are still challenging fractures. Fracture classification is the basis for understanding these injuries and to gain treatment and to choose a fracture type-based treatment concept. Using a systematic step-wise concept of fracture analysis, based on specific radiographic lines on standard X-rays allow even less experienced surgeons to get a correct classification of the elementary and associated fracture types of Letournel’s classification. Algorithmic analysis of the iliopectineal line, (anterior column involvement), ilioischial line (posterior column involvement), presence of a posterior wall fragment, fracture lines involving the iliac wing and inferior ramus, and the spur sign (representing associated both column fractures) allow for approximately 80–90% correct classifications using standard X-rays when integrating these lines into an algorithm. Especially, T-shaped fractures, ABC and ACPHT fractures may be difficult to classify. Thus, advanced imaging, such as CT scans with multiplanar reconstruction and 3D reconstructions is additionally recommended.

## Introduction

The main aim of every classification should be, that a classification should be simple and easy to use [26], giving the possibility of logical treatment decisions [12, 42, 43] and thus helping the surgeon to choose a fracture type-based treatment concept. Additionally, for the selected treatment a prognostic estimation should be possible [29].

Early classifications distinguished between posterior and central fracture (dislocations), dependent on the available treatment possibilities, especially regarding treatment of the accompanying hip dislocation [1, 4, 5, 9].

The first extended, anatomically and biomechanically based classification of acetabular fractures was proposed by Rowe and Lowell in 1961 [37]. The region of the acetabulum was anatomically divided into 3 areas, which corresponded to the primary ossification centers. Thus, an anterior pubic segment (“inner wall”), a posterior ischial segment (“posterior acetabulum”) and an iliac segment (“superior dome”) were distinguished.

The main disadvantage of these “early” classifications was, that they showed a lack of clear classification criteria.

In the early 60ies, Judet and Letournel developed the still valid and accepted classification of acetabular fractures based on a fundamental anatomical and radiological analysis of the morphology of the acetabulum [22–24].

These investigations led to a completely new understanding of acetabular fractures and is the basis of the most widely used classification systems. Anatomically, the understanding of the column principle forms this classification [24]. The acetabulum is functionally composed of two columns, an anterior and a posterior column in shape of an inverted “Y”, with the hip socket integrated at its intersection.

Maurice Müller et al. integrated the Letournel classification into their standardized and complete AO-system [29, 32, 43]. The resulting AO/OTA-classification added prognostic relevant additional injuries of the hip joint, such as marginal impaction zones, femoral head injuries and comminution zones into this “new” comprehensive classification system of acetabular fractures (Comprehensive Classification of Fractures: CCF = AO/OTA classification) [32]. The main principle is a hierarchical classification of all fractures in triple-groups. Three fracture types (A, B, C) were defined with further subdivision of three fracture groups (A1, A2, A3, B1, B2, B3, C1, C2, C3), according to its proposed increasing fracture severity. Each fracture group was further subdivided into three subgroups (A1.1–C 3.3).

In an attempt to simplify this classification, Harris et al. defined four acetabular fracture types, based on an axial CT analysis [16, 17].

**Fig. 1:**
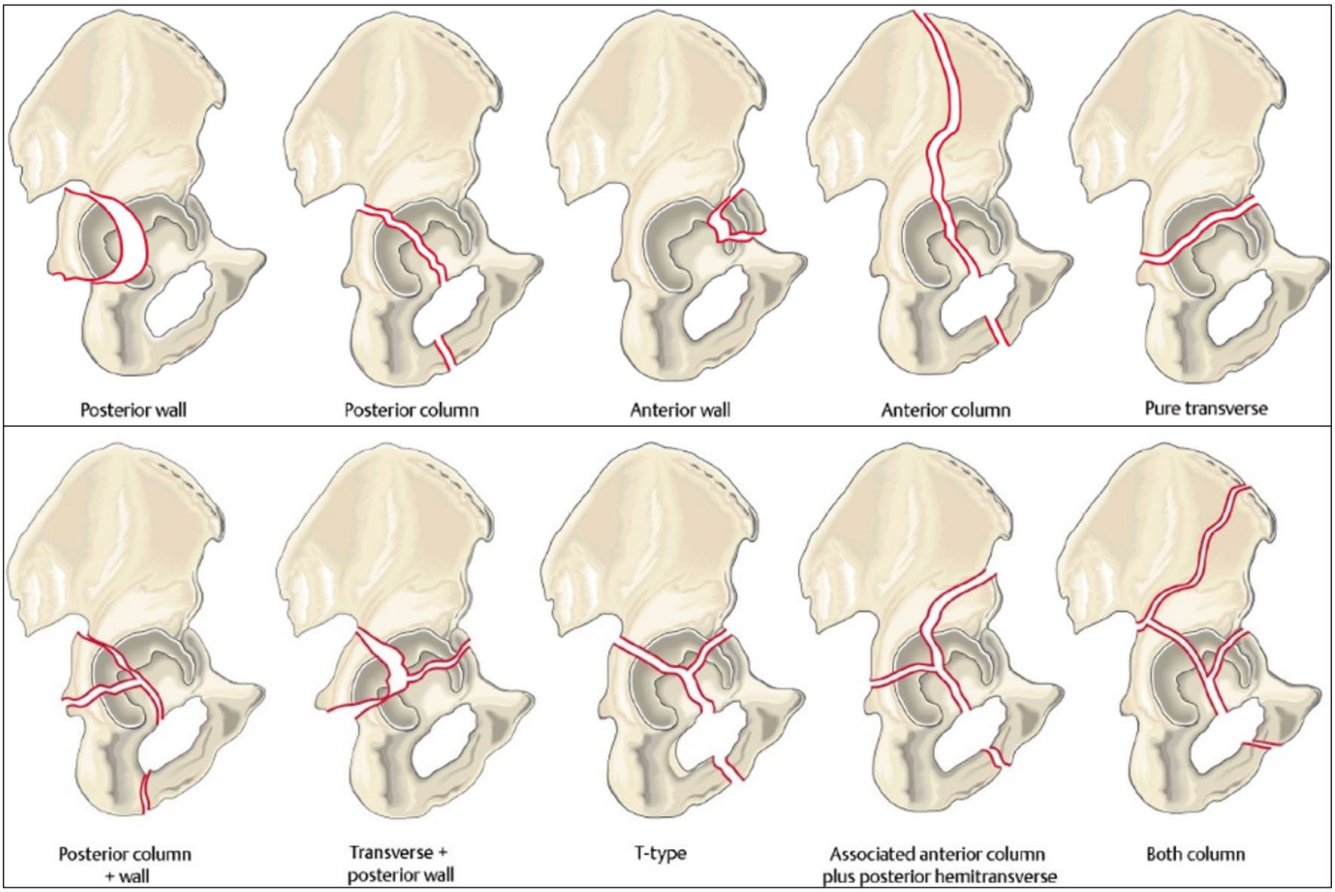
The five elementary and associated fracture types according to the Letournel classification


Ultimately, this classification is also based on the original Letournel-classification and therefore thinking in Letournel’s classic 10 fracture types remains the present basis of fracture understanding (Fig. 1).

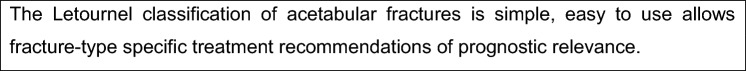


## Basis of conventional X-ray analysis

**Fig. 2:**
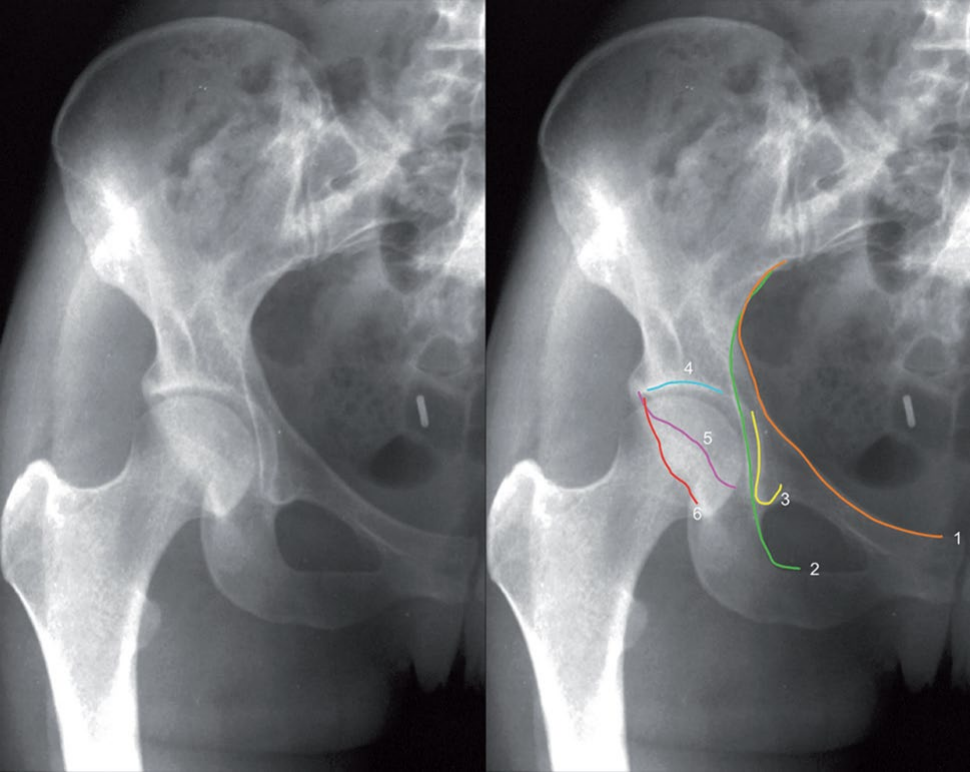
Characteristic lines on the anteroposterior view of the pelvis: 1, iliopectineal line; 2, ilioischial line; 3, tear drop figure; 4, acetabular roof; 5, anterior wall line 6, posterior wall line

The pelvic AP view is still the gold standard in the evaluation of acetabular fractures. According on Letournel’s detailed anatomical and radiographic analysis six lines are relevant for fracture classification (Fig. 2) [24]:

- iliopectineal line = represents to ¾ the medial part of the anterior column; disruption indicates anterior column involvement
- ilioischial line ilioischial line represents the posterior column in its lower and middle parts; disruption indicates posterior column involvement
- posterior wall line = usually a slightly S-shaped line and most lateral border of the acetabulum due to the physiological anteversion of the acetabulum (consider: acetabular retroversion, crossing sign [45]); disruption indicates a fracture at the posterior wall (not necessarily a posterior wall fracture)
- anterior wall line = double S-shaped line starting at the anterior and superior margin of the obturator foramen, typically medial to the posterior wall line; disruption indicates a fracture at the anterior wall (not necessarily an anterior wall fracture)
- acetabular roof line = represents the major weight-bearing area without defining its extent when disrupted
- teardrop figure = not an antomical, but a radiographic feature; corresponds to parts of the lateral (acetabular fossa) and medial wall (distal quadrilateral surface and more proximal obturator canal; disruption indicates distal quadrilateral plate involvement and can show femoral head displacement

In addition to these lines, analysis of the pelvic AP view includes serach for additional iliac fossa fracture lines extending up to the iliac crest, as typically seen in fractures with a high anterior column component. Inferior pubic ramus fractures and posterior wall fragment (lateral and superior to the acetabular border have to be analyzed [14].

### Judet views (iliac and obturator oblique views)

As no true radiological perpendicular plane is available, Judet and Letournel defined hemipelvic oblique views considering the morphological anatomy of the hemipelvis, that the iliac wing/fossa is rotated 90°in relation to the obturator segment.

**Fig. 3:**
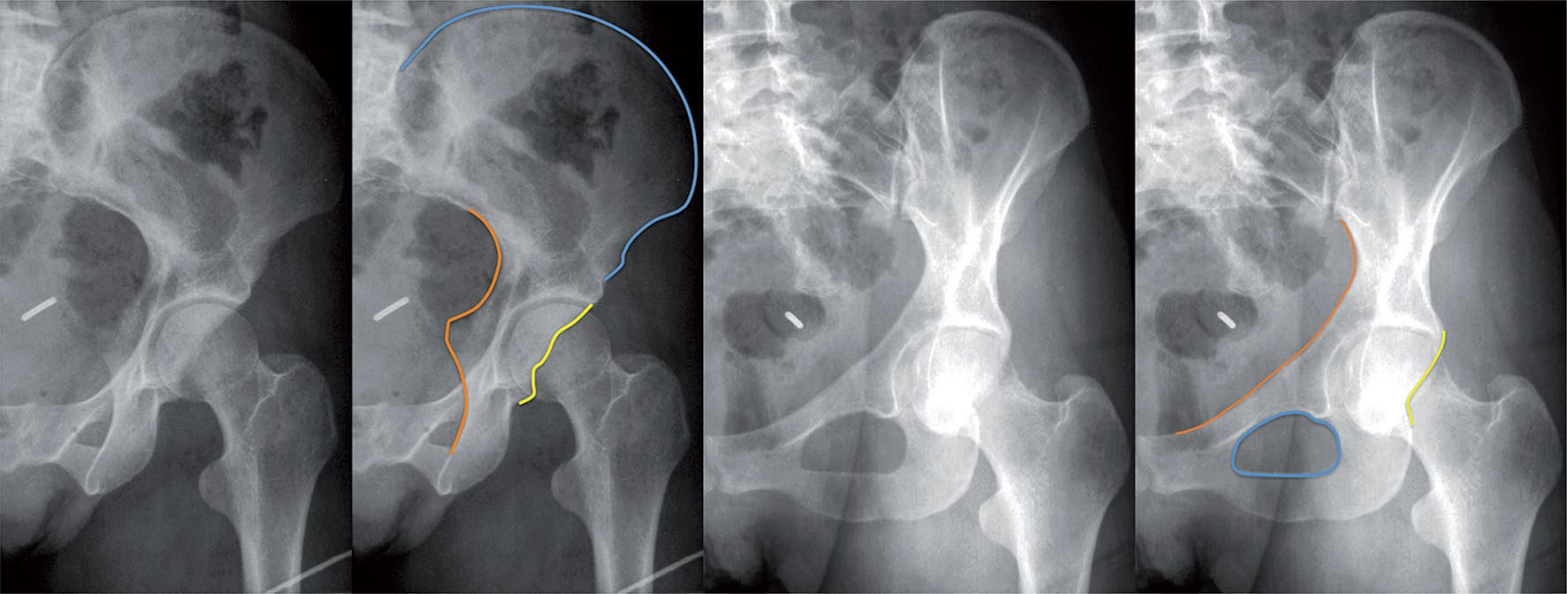
Characteristics of the iliac oblique view (left two X-rays). Illustration of the lines of the posterior column (orange), the anterior wall (yellow), and the iliac fossa (blue). Characteristics of the obturator oblique view (right two X-rays). Illustration of the lines of the anterior column (orange), the posterior wall (yellow), and the obturator foramen (blue)

Thus, the iliac oblique view (Fig. 3) allows analysis of the iliac fossa, the posterior column, and the anterior wall, but not the obturator segment, while the obturator oblique view (Fig. 3) allows analysis of the obturator foramen, the anterior column, and the posterior wall [14].

### Basic CT-analysis

CT of acetabular fractures is crucially important in providing detailed analysis of intraarticular lesions and additionally allows for easier differentiation of certain fracture types.

**Fig. 4:**
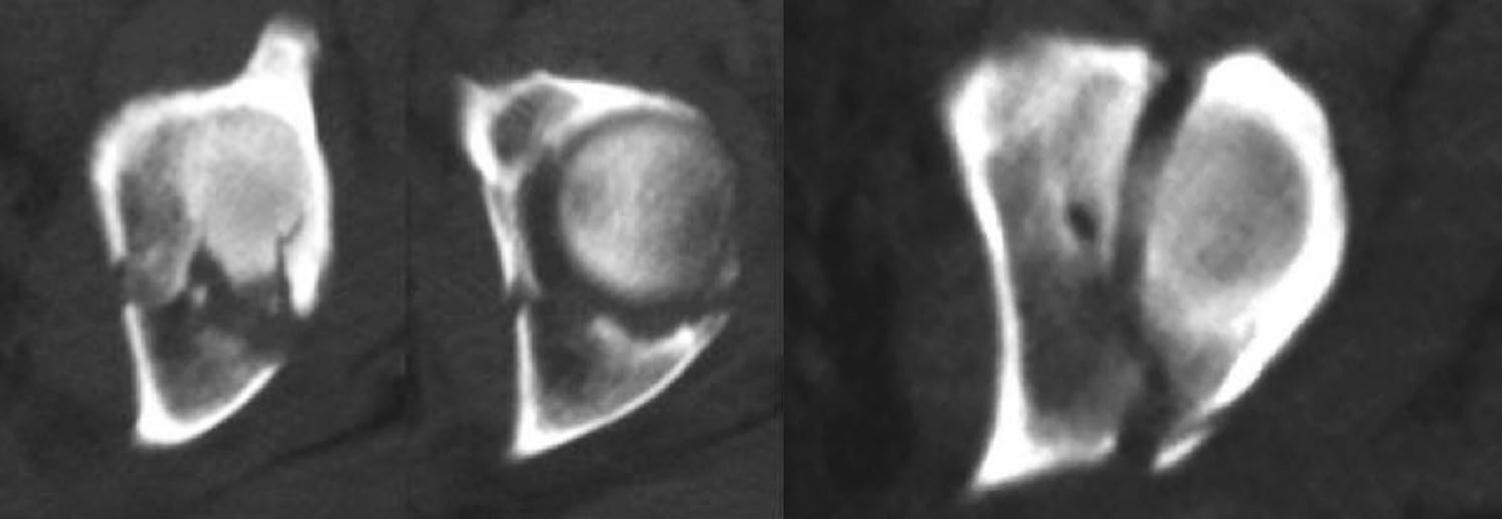
Typical column separation, indicated by a “transverse” fracture line on axial CT (left two CT-views) and a typical “vertical” fracture line indicated by a transverse fracture with the medial fragment being unstable (right CT-view)

**Fig. 5:**
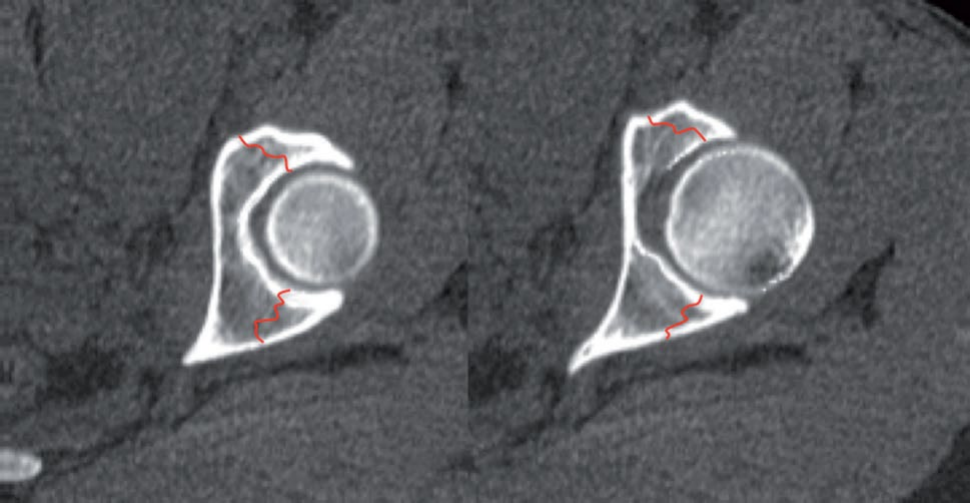
Typical orientation of fractures of the anterior and posterior wall

The basis of CT-analysis is the axial plane. A detailed description of CT-analysis is performed elsewhere. The relevant conclusions from axial CT analysis are as follows:

- the anterior column is seen anteriorly on axial CT and the posterior column is located posterior; column separation is represented by a horizontal fracture line; anterior or posterior column displacement/instability represents anterior or posterior column fracture, while anterior and posterior displacement indicates anterior and posterior column instability (Fig. 4a)
- a “vertical” fracture line indicated a transverse fracture component; it is important to analyze all consecutive images, and not only distal image planes, to avoid misinterpretation of anterior and posterior fracture lines at the level of the walls; the medial fragment is always the unstable fragment, as the obturator segment is typically displaced and rotated into the true pelvis around a vertical axis through the pubic symphysis (Fig. 4b)
- wall fractures present in a more oblique orientation compared to the distal transverse fracture component (Fig. 5)

**Fig. 6:**
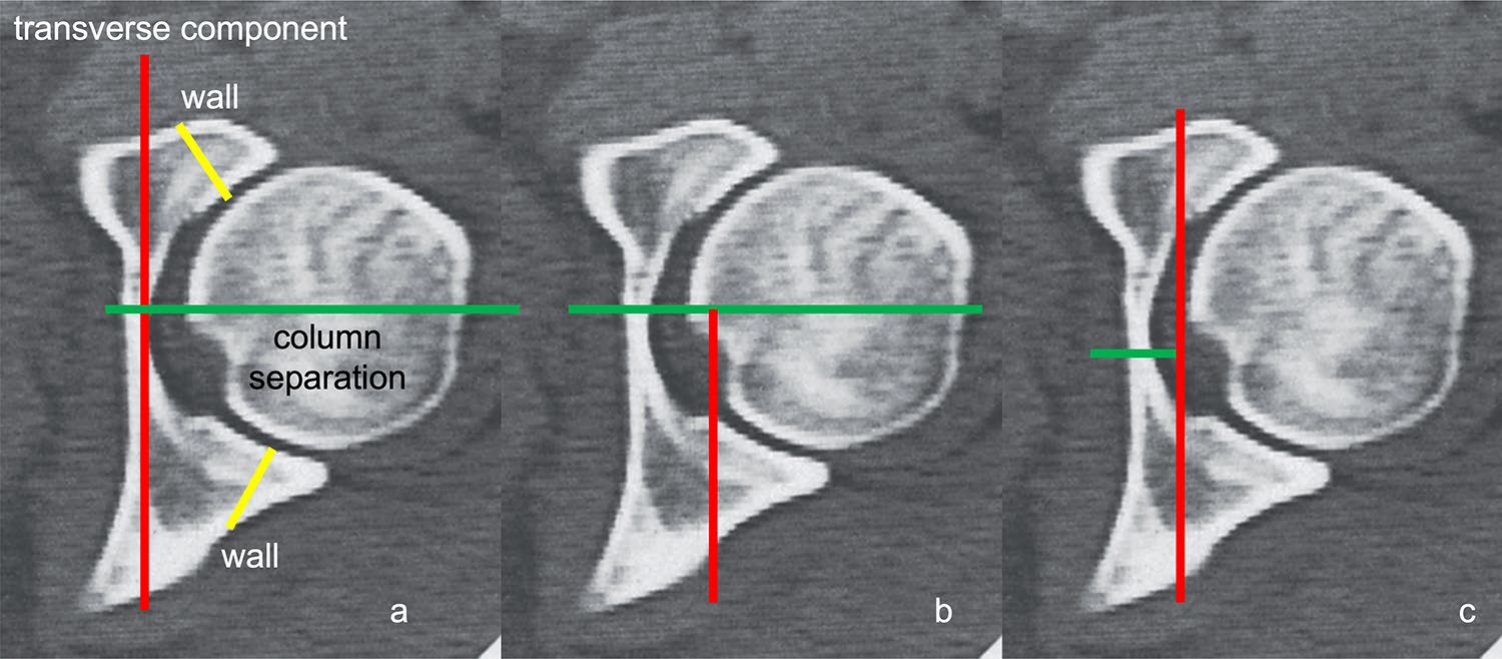
Basic fracture orientations on axial CT-views (**a**). Typical T-letter indicating and ACPHT fracture (**b**) and rotated T-letter in T-shaped fractures (**c**)

Using these lines, the typical simple, elementary fracture types can be easily detected. Combining these basic fracture lines on the axial CT allow distinguishing several associated fracture types, especially safe differentiation between associated both column fractures (ABC), associated anterior column plus posterior hemitransverse fractures (ACPHT), and T-shaped fractures (Fig. 6):

- ABC = analysis of the remaining ilium fragment connected to the SI-joint/sacrum from proximal to distal; if no articular part is connected an ABC fracture is proven, while an articular connection indicates an ACPHT fracture; ABC definition: complete separation of both columns on axial CT images with a transverse main fracture line and no articular fragment connected to the SI-joint
- ACPHT = primary horizontal fracture line (anterior column separation or displacement) and a typical transverse fracture exclusively in the posterior part the acetabulum (posterior “vertical” fracture = hemitransverse component) showing a T-letter
- T-shaped = primary transverse fracture line plus a distal column separation (“horizontal” fracture line starting from the “vertical” line and running medially) showing a 90° rotated T-letter in left-sided fractures and a 90° left-rotated T is-letter in right-sided fractures

## Reliability analyses

Various authors analyzed the reliability of Letournel’s acetabulum fracture classification.

### Value of conventional X-rays

Beaulé et al. revealed a significant increase of consistency with increasing experience, but no significant difference was found between experts and very experienced surgeons analyzing conventional X-rays [2]. Overall, a good agreement was observed between raters. However, this group of examiners represents an expert group as even the most inexperienced investigators operated up to 50 acetabular fractures within 5–10 years. In a further interobserver reliability analysis, results were poor analyzing only conventional X-rays compared to examinations with additional axial CT or 3D-CT [30]. Comparing 3D views and Judet views showed a low agreement level [41].
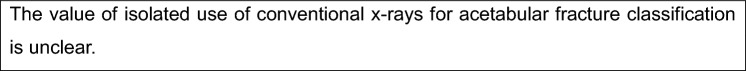


### Value of Judet views (iliac and obturator oblique views)

Analyzing conventional X-ray sets (pelvic a.p. view, Judet views) revealed, that best agreement was observed when analyzing the iliopectineal line, the ilioischial line and the presence of a posterior wall fragment, whereas the Judet-views had no influence on improvement of agreement. Overall, an experience dependent agreement was observed [33]. Similar results were reported comparing conventional radiographs and CT scans with better results (good agreement) after CT diagnostics [31].

In an analysis of conventional X-rays with additional 2D CT vs. adding 3D views, the interobserver agreement was moderate for all image sets, with better results not using the oblique (Judet) views [10].
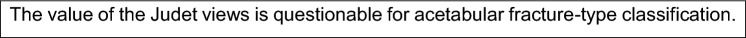


### Value of CT-images

Hüfner et al. observed, that experienced examiners were more likely to benefit from conventional 2D-CT diagnostics, and less experienced examiners improved their classification skills [18].

The importance of CT examination with or without 3D-images, in combination with conventional radiographs was elaborated recently. Interobserver reliability was significantly better analyzing only conventional X-rays compared to examinations with additional axial CT or 3D-CT. Interestingly, analysis of conventional views generated from CT data showed identical results compared to pure CT analyzes [30].

Hurson et al. performed an inter- and intraobserver analysis based on conventional X-rays and CT images as well as of bone models generated from the CT data set [19]. Consultants and residents showed better results using these models compared to conventional radiographs (k = 0.76 vs. 0.51 and k = 0.71 vs. 0.42, respectively).

2D CT-files including multiplanar reconstructions led to 56.7% accuracy of classification [39]. Adding of four standardized 3D views increased the interobserver reliability.
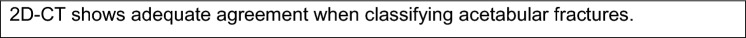


### Value of 3D images

Visutipol et al. first compared the relevance of 3D illustration of acetabular fractures with conventional radiographs [44] and observed only a very low to moderate interobserver reliability. 3D-images showed no advantage in classifying acetabular fractures.

A recent analysis showed a near perfect agreement level between 3D-images and intraoperative classification, while conventional X-rays showed an agreement close to chance [41]. 3D-CT-files were superior to 2D-files with multiplanar reconstructions and led to 73% accuracy of classification [39].

In an analysis of only five common fracture types (posterior wall fracture, 4 associated fracture types) a 3DCT set of 39 images was superior to 2DCT images in terms of diagnosis and speed of classification. A profit was observed, especially analyzing transverse- and posterior wall-type fractures [20].
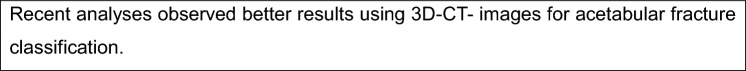


### Value of 3D models

Hurson et al. performed an inter- and intraobserver analysis based on conventional X-rays and CT images as well as of 3D bone models generated from the CT data set [19]. Consultants and residents showed better results using these models compared to conventional radiographs (k = 0.76 vs. 0.51 and k = 0.71 vs. 0.42, respectively). The relevance of integrating a 3D-model was confirmed by Hansen et al., who observed better accuracy in classifying acetabular fractures [15].

3D models improved the classification accuracy compared to the standard conventional X-ray set and allowed even trainees to adequately classify acetabular fractures [25].

Brouwers et al. found 3D-virtual reality inferior to 3D printing models in classifying acetabular fractures [7].

Analyzing only seven patients with CT images and with corresponding 3-D printed models showed, that 3D models increased the inter-observer agreement rates for fracture classification, but decreased it with respect to the preferred surgical approach [21].
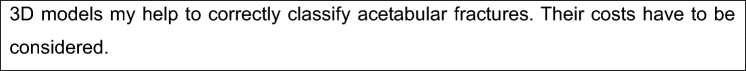


### Fracture type analysis

Specific fracture types, e.g. T-type fractures, associated both column fractures (ABC), anterior wall fractures, and associated anterior column plus posterior hemitransverse fractures (ACPHT) seem to be difficult to classify [2, 14].
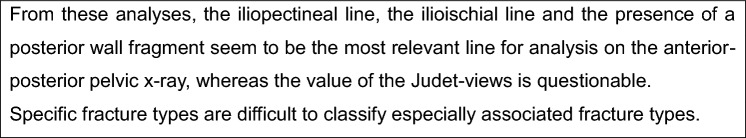


## Algorithms for fracture classification

Already in 1998, Brandser et al. proposed a simplification of acetabular fracture analysis [6]. A systematic approach with mandatory CT examination was proposed distinguishing between fractures with involvement of an acetabular wall, acetabular column and transverse fracture component followed by division into the 10 fracture types according to Letournel. Only by using all 4 modalities (pelvis a.p. x-ray, ilia oblique and obturator oblique view, CT), a safe classification was possible. A focus was set on fracture lines involving the obturator foramen, the iliopectineal and/or ilioischial line, the iliac wing and the presence of a posterior wall fracture and/or a “spur-sign” [6].

Durkee et al. proposed an algorithm focusing more on these relevant lines for acetabular fracture classification [11]. The presented algorithm using these lines clearly separated 5 of the 10 Letournel fracture types. However, the other fracture types remained unconsidered.

Adding the disruption or integrity of the obturator ring as a criteria to such an algorithm, lead to an improvement of consistency rates from 59.9 to 71.2% [35].

Since the late 1990s, Tim Pohlemann developed a classification algorithm, based on standard X-rays, with analysis of the iliopectineal line, ilioischial line, presence of a posterior wall fragment and d/or disruption of the obturator foramen. The resulting algorithm was tought on several pelvic fracture management courses (e.g. AO Trauma Course 2005). Gänsslen et al. published this algorithm in 2015 and included a CT analysis for T-shaped fractures and associated anterior column + posterior hemitransverse fractures [13, 14] (Fig. 1).

In contrast non-specialist/non-experienced experienced that methodical training in the interpretation of radiographs was not effective [34].

Several authors published fracture classification algorithms, the majority based on standard rediographs.

One of the first papers dealing with such an algorithm was introduced by Ly et al. [27] together with three highly active and experienced pelvic and acetabular surgeons. The sequence of analysis of this algorithm included analysis of:

- disruption of the iliopectineal or ilioischial lines
- number of involved columns
- presence of ischial ramus fracture
- presence of a posterior wall fragment
- involvement of the iliac wing
- presence of a spur sign

Using this algorithm led to an improvement of correctly classified acetabular fractures (15 cases, all 10 Letournel fracture types) from 50 to 59%. As 41% were still incorrectly classified, it was stated, that “this or other such algorithms could provide residents with a basic tool to better evaluate standard radiographs and classify acetabular fractures” [27]. Medical students showed an increase of accuracy from 20 to 41.5% [8].

An 3D-based algorithm developed by radiologists and orthopedic surgeons included analysis of a posterior wall fragment, involvement of the superior acetabulum, involvement of the anterior and posterior portions of the acetabulum, without analyzing reliability results [38].

Mauffrey et al. first analyzed the disruption of either the iliopectineal and/or ilioischial line [28]. Based on the involvement, the next step included analysis of the presence of a posterior wall or anterior fragment (only if both lines were undisrupted) or the presence of an intact or disrupted obturator ring segment. The 3rd step analyzed the iliac wing for presence of a fracture or the presence of the spur-sign, the latter confirming an associated both column fracture. Only if the obturator ring was intact, again the presence of posterior wall fragment was analyzed.

Riouallon et al. created an app with a classical sequence for fracture analysis based on CT data [36]:

1. involvement of the iliac wing (fracture)
2. ischial spine fracture (e.g. inferior pubic ramus fracture)
  3.1 articular fragment attached to the SI-joint
  3.2 fracture of the obturator ring
  3.3 independent posterior wall fragment
3. iliopectineal line disruption
4. independent posterior wall fragment

Compared to 2D-CT scans with multiplanar reconstructions, using the app increased accuracy of fracture classification from 64.5 to 83.4%. Especially, young surgeons showed the greatest profit of improvement with an excellent reliability [36]

A 2D-CT analysis used a sequence with first analyzing the presence of an inferior pubic ramus fracture, followed by analysis of the presence of column fracture(s), but without presenting data of reliability [3].

In a recent analysis, classification was performed using an algorithm with only §d imaging [40]. The algorithm was based on several questions:

- Are there fractures to the anterior or posterior column?
- Is there a posterior wall fracture? or Is there a fracture of the posterior column?
- Is there a transverse fracture line? or Is there anterior column fracture?
- Is there involvement of the obturator ring? or Is there a posterior wall fracture? or Is there a spur?

An increase from 52.5 to 77.5% accuracy was observed when using this algorithm compared to analysis without the algorithm [40].
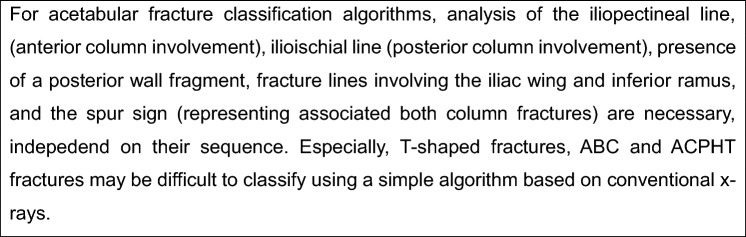


## Proposed classification algorithm

The aim of any algorithm is to sequentially confirm or exclude fracture types. Based on experience from the last two decades, starting with the well established lines on the AP view, e.g. the iliopectineal (ip) and ilioischial line (ii), a first and simple distinction can be made. Considering the elementary and associated fracture types, described by Letournel, e.g. posterior wall (PW), posterior column (PC), anterior wall (AW), anterior column, pure transverse (pT), associated posterior column and posterior wall (PC/W), transverse plus posterior wall (TPW), T-shaped (T), ACPHT and ABC, initial analysis of these lines is advantageous (Table 1):

- isolated iliopectineal line disruption (iip) = isolated anterior column involvement → excludes all fracture types with posterior involvement (PW, PC, PC/W, pT, TPW, T, ACPHT, ABC)
- isolated ilioischial line disruption (iii) = isolated posterior column involvement → excludes all anterior fracture types (AW, AC)
- combined iliopectineal (ip) and ilioischial (ii) line disruption = involvement of the anterior and posterior column (e.g. pT, TPW, T, ACPHT, BC) → excludes pure anterior (AW, AC) nd pure posterior (PW, PC, PC/W) fracture types

**Table 1:** Analysis of disruption of the iliopectineal and/or ilioischial line and remaining or excluded fracture types

Disrupted line(s)	Possible fracture types	Excluded fracture types
isolated iliopectineal	AW, AC	PW, PC, PC/W, pT, TPW, T, ACPHT, ABC
isolated ilioischial	PW, PC, PC/W	AW, AC, pT, TPW, T, ACPHT, ABC
iliopectineal + ilioischial	pT, TPW, T, ACPHT, BC	AW, AC, PW, PC, PC/W

A possible next relevant line cam be the fracture of the inferior ramus, which is nearly always present in fracture involving the anterior common. Based on the above decribed analysis of the intactness or disruption of the iliopectineal or ilioischial line, an inferior ramus fracture is usually present in AC, PC, PC/PW, T-shaped, ACPHT and ABC fractures, while the inferior ramus is usually intact in AW, PW, pT, and T/PW, respectively. This lead to further differentiation according to Table 1 (Table 2).

**Table 2:** Additional analysis of disruption of the iliopectineal and/or ilioischial line and disruption or intactness of the inferior pubic ramus showing the remaining or excluded fracture types

Disrupted line(s)	Possible fracture types	Excluded fracture types
isol. iliopectineal + inferior ramus	AC	*AW*
isol. ilioischial + inferior ramus	PC, PC/W	*PW*
ip + ii + inferior ramus	T, ACPHT, ABC	pT, TPW
isol. iliopectineal − inferior ramus	*AW*	*AC*
isol. Ilioischial − inferior ramus	*PW*	PC, PC/W
ip + ii − inferior ramus	pT, TPW	T, ACPHT, BC

Already diagnosed fracture type (italics)

At this point, PW, AW and AC fractures are already diagnosed (row 1, 4, 5). If a typical posterior wall fragment can be observed, which is present in pure PW, PC/W and TPW fractures, a clear differentiation can be made in row 2 and 6 of Table 2 (Table 3):

- if a PW fragment is present according to row 2 a PC/W fracture is diagnosed, while an isolated PC fracture is excluded
- if PW fragment is present according to row 6 a TPW fracture is diagnosed, while a pure transverse fracture is excluded

**Table 3:** Analysis of row 2 and 6 from Table 2: a distinction can be made between PC/W and isolated PC fractures (former row 2) and between pT and TPW fractures (former row 6)

Disrupted line(s)	Possible fracture types	Excluded fracture types
isol. ilioischial + inferior ramus + PW	PC/W	PC
ip + ii − inferior ramus + PW	TPW	pT

Some fracture types show fracture lines extending between the anterior inferior iliac spine (AIIS) up to the iliac crest. These fracture types represent iliac fossa involvement, which is usually seen in AC, ACPHT and ABC fractures. The remaining row 3 from Table 2 left these three fracture types. Only T-shaped fractures usually have no iliac fossa involvement. Thus, if no fracture line extends into the iliac wing, a T-shaped fracture is likely.

The remaining two fracture types, ACPHT and ABC fracture, are sometimes difficult to diagnose on the AP view of the pelvis. The pathognomonic feature of an ABC fracture is the spur sign, which can be optimally seen on the obturator oblique view. This spur corresponds to the iliac fragment attached to the SI-joint without having any articular fragment attached. Thus, the spur sign distinguishes between ACPHT and ABC fractures.

**Fig. 7:**
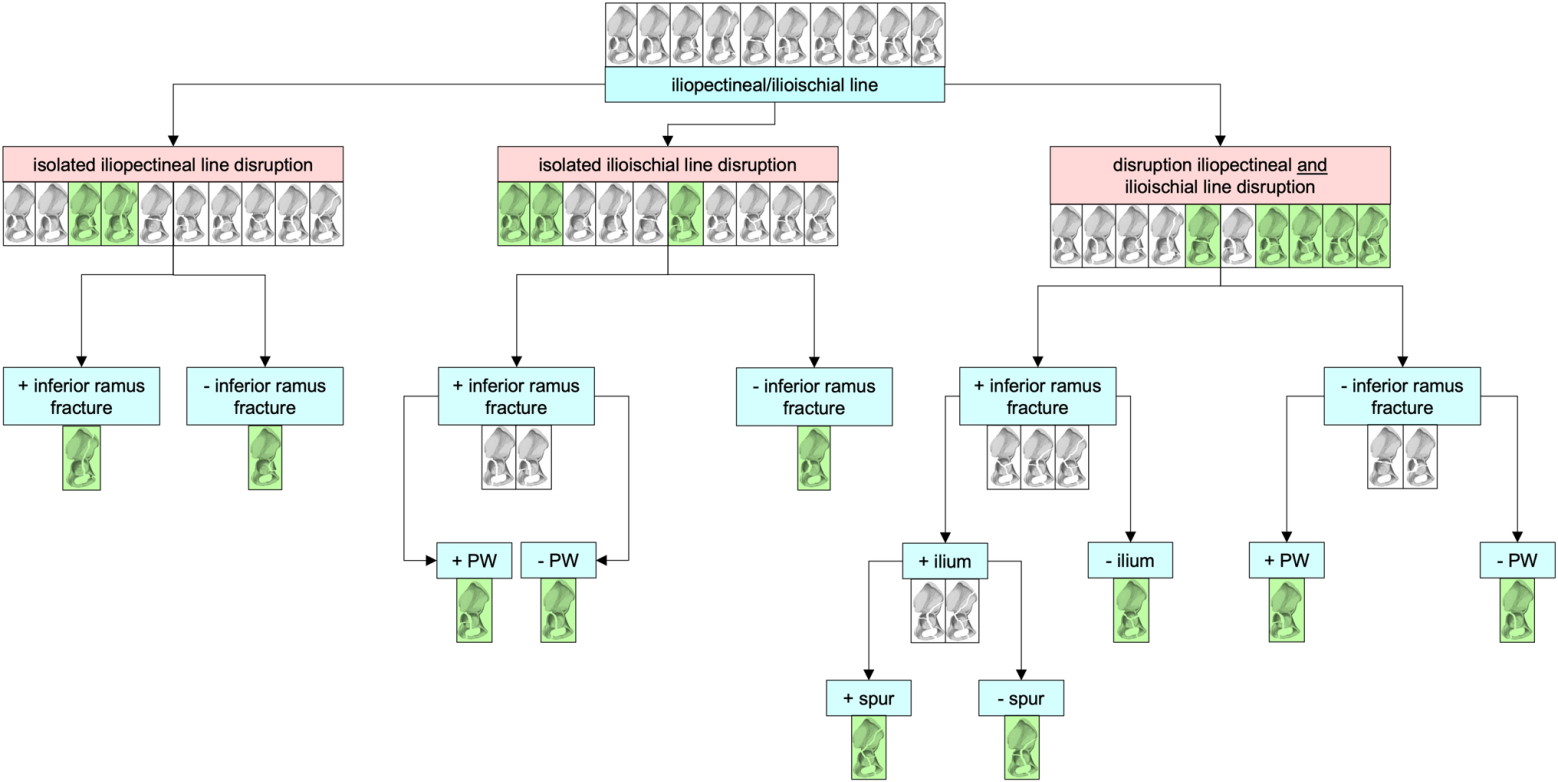
Possible and recommended algorithm for classification of acetabular fractures based on typical fracture line characteristics

Putting this information into a sequential algorithm, makes fracture analysis much easier (Fig. 7).

## Conclusion

Acetabular fractures are still challenging fractures. The gold-standard of classifying these fractures is based on Letournel’s anatomic and radiological work. This classification consists of five elementary and five associated fracture types. Letournel defined several radiographic lines corresponding to specific fractures.

In general, a classification should be simple and easy to use, giving the possibility of logical treatment decisions and thus helping the surgeon to choose a fracture type-based treatment concept. Additionally, a prognostic estimation should be possible.

Acetabular fracture classification should be systematically performed in a stepwise fashion. Several reports dealed with algorithms to allow a simple classification. Based on published algorithms, analysis of the iliopectineal line, (anterior column involvement), ilioischial line (posterior column involvement), presence of a posterior wall fragment, fracture lines involving the iliac wing and inferior ramus, and the spur sign (representing associated both column fractures) are necessary, independent on their sequence. Using algorithms allow for approximately 80–90% correct classifications using standard X-rays. Especially, T-shaped fractures, ABC and ACPHT fractures may be difficult to classify. Thus, advanced imaging, such as CT scans with multiplanar reconstruction and 3D reconstructions is additionally recommended.

Algorithms lead to a conventional radiographic understanding of acetabular fractures, which is also of intraoperative relevance.
